# Can Sulfate Be the First Dominant Aqueous Sulfur Species Formed in the Oxidation of Pyrite by *Acidithiobacillus ferrooxidans*?

**DOI:** 10.3389/fmicb.2018.03134

**Published:** 2018-12-18

**Authors:** Sarka Borilova, Martin Mandl, Josef Zeman, Jiri Kucera, Eva Pakostova, Oldrich Janiczek, Olli H. Tuovinen

**Affiliations:** ^1^Department of Biochemistry, Faculty of Science, Masaryk University, Brno, Czechia; ^2^Department of Geological Sciences, Faculty of Science, Masaryk University, Brno, Czechia; ^3^Department of Microbiology, The Ohio State University, Columbus, OH, United States

**Keywords:** *Acidithiobacillus ferrooxidans*, cellular ATP, pyrite electrode, pyrite oxidation, tetrathionate hydrolase, iron-oxidizing bacteria

## Abstract

According to the literature, pyrite (FeS_2_) oxidation has been previously determined to involve thiosulfate as the first aqueous intermediate sulfur product, which is further oxidized to sulfate. In the present study, pyrite oxidation by *Acidithiobacillus ferrooxidans* was studied using electrochemical and metabolic approaches in an effort to extend existing knowledge on the oxidation mechanism. Due to the small surface area, the reaction rate of a compact pyrite electrode in the form of polycrystalline pyrite aggregate in *A. ferrooxidans* suspension was very slow at a spontaneously formed high redox potential. The slow rate made it possible to investigate the oxidation process in detail over a term of 100 days. Using electrochemical parameters from polarization curves and levels of released iron, the number of exchanged electrons per pyrite molecule was estimated. The values close to 14 and 2 electrons were determined for the oxidation with and without bacteria, respectively. These results indicated that sulfate was the dominant first aqueous sulfur species formed in the presence of bacteria and elemental sulfur was predominantly formed without bacteria. The stoichiometric calculations are consistent with high iron-oxidizing activities of bacteria that continually keep the released iron in the ferric form, resulting in a high redox potential. The sulfur entity of pyrite was oxidized to sulfate by Fe^3+^ without intermediate thiosulfate under these conditions. Cell attachment on the corroded pyrite electrode surface was documented although pyrite surface corrosion by Fe^3+^ was evident without bacterial participation. Attached cells may be important in initiating the oxidation of the pyrite surface to release iron from the mineral. During the active phase of oxidation of a pyrite concentrate sample, the ATP levels in attached and planktonic bacteria were consistent with previously established ATP content of iron-oxidizing cells. No significant upregulation of three essential genes involved in energy metabolism of sulfur compounds was observed in the planktonic cells, which represented the dominant biomass in the pyrite culture. The study demonstrated the formation of sulfate as the first dissolved sulfur species with iron-oxidizing bacteria under high redox potential conditions. Minor aqueous sulfur intermediates may be formed but as a result of side reactions.

## Introduction

The oxidation of pyrite by bacteria contributes to microbial sulfur cycling in the environment. As pyrite is the most common and widely spread metal sulfide in the environment, knowledge of the mechanism of its oxidation and related pathways is fundamentally significant in the biochemistry and physiology of acidophilic iron- and sulfur-oxidizing bacteria and in pyrite biogeochemistry. Pyrite oxidation is one of the key biological reactions in exposed sulfide mineral deposits and particularly relevant in the bioleaching of metals from low-grade ores or concentrates (Schippers et al., 2014; Banerjee et al., 2017; Sethurajan et al., 2018; Werner et al., 2018). Characterization of its oxidation mechanism provides a basic understanding of the substrates, products and enzyme systems that should be considered to control the overall process. *Acidithiobacillus ferrooxidans* is a widely used model of acidophilic iron- and sulfur-oxidizing bacteria that are involved in these processes although many other acidophiles can also participate in various oxidation steps (Johnson, 2012; Hedrich et al., 2016; Nuñez et al., 2017; Quatrini and Johnson, 2018). Pyrite is virtually ubiquitous in sulfide mineralizations and often plays a fundamental role in sulfur biogeochemistry in natural and bioleaching impacted environments. Its bacterial oxidation results in the formation of ferric iron and sulfuric acid as ultimate end products. Ferric iron is an important chemical oxidant for dissolution of sulfide minerals under acidic conditions. Pyrite oxidation in exposed sulfide mineralizations is responsible for acid mine drainage and solubilization of metals from minerals (Wu et al., 2013; Blowes et al., 2014; Dold, 2014; Hipsey et al., 2014; Zheng et al., 2017). In addition to the toxic effects of sulfuric acid and heavy metals, non-specific pH-dependent phytotoxic effects of ferric iron in the form of Fe(III)-precipitates can interfere with plant physiological processes (Bartakova et al., 2001).

The acid-yielding process of the bacterial and chemical pyrite oxidation has been known at least since the mid-1900’s, and the basis of the oxidation mechanism – i.e., which intermediates may be formed and their role in the process – has also been in focus for decades. The thiosulfate mechanism is currently widely accepted. The complete oxidation of the pyrite sulfur entity by ferric iron (Equation 1) is described as follows (Schippers et al., 1999):

(1) $${\mathrm{FeS}}_{2}+14{\mathrm{Fe}}^{3+}+8H_{2}O\to 15{\mathrm{Fe}}^{2+}+2{\mathrm{SO}}_{4}{}^{2-}+16H^{+}$$

Pyrite oxidation by oxygen under acidic conditions is much slower and is usually not considered in the presence of ferric iron. Under acidic conditions, Fe^2+^ thus formed is re-oxidized to Fe^3+^ by iron-oxidizing bacteria (Equation 2) to promote further chemical pyrite oxidation:

(2) $$4{\mathrm{Fe}}^{2+}+O_{2}+4H^{+}\to 4{\mathrm{Fe}}^{3+}+2H_{2}O$$

The extent of free acidity from pyrite oxidation depends on reactions such as ferrous iron oxidation that compete for protons. Further acidification is produced by precipitation of Fe(III)-hydroxysulfates such as schwertmannite and various jarosites, which are commonly found in acidic environments impacted by pyrite or iron oxidation (Bigham and Nordstrom, 2000; Kaksonen et al., 2014).

Thiosulfate has been postulated as the first soluble intermediate of pyrite oxidation mediated by Fe^3+^. In this scheme, the pyrite surface catalyzes thiosulfate oxidation by ferric iron to tetrathionate, which is further oxidized to sulfate through other sulfur intermediates (Schippers et al., 1996, 1999, 2014; Schippers and Sand, 1999; Rohwerder et al., 2003; Vera et al., 2013). Moses et al. (1987) proposed a reaction mechanism with thiosulfate as the first product of abiotic pyrite oxidation by Fe^3+^ and oxygen at pH between 2 and 9. Molecular orbital theory consideration also supported thiosulfate as the first sulfur intermediate (Luther, 1987). Tetrathionate, in addition to thiosulfate and some other intermediates, was proposed to be formed from thiosulfate (Descostes et al., 2004). Some intermediate sulfur oxyanion species may be formed at very low concentrations but, because of their instability at low pH values, they may not be readily detected. Thiosulfate (S_2_O_3_^2-^), trithionate (S_3_O_6_^2-^), tetrathionate (S_4_O_6_^2-^), and disulfane-monosulfonic acid (S_3_O_3_^2-^) have been considered as key intermediate sulfur compounds in the oxidation of pyrite. The concept of the thiosulfate mechanism was based on the detection of sulfur intermediates (polythionates at micromolar concentrations) during pyrite oxidation by *Leptospirillum ferrooxidans* (no sulfur-oxidizing activity), *A. ferrooxidans*, acidic ferric sulfate solution, and under alkaline conditions (Schippers et al., 1996). This concept was developed further to construct the proposal on the thiosulfate mechanism of pyrite oxidation by acidophilic iron- and sulfur oxidizers. The following reactions were considered in this pathway (Schippers et al., 1996, 2014; Schippers and Sand, 1999):

(3) $${\mathrm{FeS}}_{2}+6{\mathrm{Fe}}^{3+}+3H_{2}O\to 7{\mathrm{Fe}}^{2+}+S_{2}O_{3}{}^{2-}+6H^{+}$$

(4) $$2S_{2}O_{3}{}^{2-}+2{\mathrm{Fe}}^{3+}\to S_{4}O_{6}{}^{2-}+2{\mathrm{Fe}}^{2+}$$

(5) $$S_{4}O_{6}{}^{2-}+H_{2}O\to {\mathrm{SO}}_{4}{}^{2-}+S_{3}O_{3}{}^{2-}+2H^{+}$$

Formation of S_3_O_3_^2-^ is deduced by its reaction products but cannot be analytically confirmed. This highly reactive disulfane-monosulfonic acid may decompose to elemental sulfur and sulfite (sulfite is oxidized by Fe^3+^ or O_2_ to sulfate).

S_2_O_3_^2-^ may also be formed by reaction of S_3_O_3_^2-^ with S_4_O_6_^2-^. Other reactions of S_3_O_3_^2-^ with S_2_O_3_^2-^, S_4_O_6_^2-^, O_2_ or Fe^3+^ may form a mixture of S_5_O_6_^2-^ (pentathionate), S_2_O_3_^2-^, S_8_ (elemental sulfur) and S_3_O_6_^2-^ and ultimately form SO_4_^2-^ as the end product by subsequent biotic and/or abiotic oxidation reactions. The exopolymer layer containing Fe^3+^ may provide a reaction compartment for the above mentioned reactions in *A. ferrooxidans* attached on pyrite surface (Gehrke et al., 1998). From the point of view of the thiosulfate mechanism and the role of acidophilic bacteria, iron- and sulfur-oxidizing activities are needed to oxidize Fe^2+^ to Fe^3+^ and the intermediate sulfur entities to sulfate. As tetrathionate is soluble and relatively stable in acid solutions, the activity of the tetrathionate hydrolase TetH seems to be especially important in the pyrite culture for tetrathionate utilization by attached and planktonic bacteria to form further intermediates and sulfate. However, tetrathionate hydrolysis as well as the other reactions of sulfur intermediates in pyrite oxidation are considered to take place under both biotic and abiotic conditions (Schippers et al., 1996). Investigation of the isotopic composition of sulfate formed during pyrite oxidation showed that 12.5% of the oxygen in sulfate was derived from ^18^O_2_ and the rest from water (Balci et al., 2007). This was ascribed to the oxidation of the above intermediate sulfite to sulfate by dissolved oxygen, thus to be consistent with the reaction scheme of the pyrite oxidation according to the thiosulfate mechanism. In addition to S^0^, S_3_O_6_^2-^, S_4_O_6_^2-^, and S_5_O_6_^2-^, hexathionate S_6_O_6_^2-^ was detected at micromolar concentrations and elemental sulfur accumulated on the surface of pyrite residues (Tu et al., 2017a). Thus, a wide spectrum of sulfur compounds may be formed during bacterial pyrite oxidation.

Based on electrochemical properties of the reaction mechanism, thiosulfate is released into the solution at higher pH and sulfate at low pH, and cations representing soft bases increase the process of sulfate formation. As summarized by Rimstidt and Vaughan (2003), aqueous pyrite oxidation involves a spectrum of soluble sulfur compounds, with almost 100% sulfate in low pH solutions and substantial concentrations of thiosulfate and its intermediate oxidation products at higher pH. The disulfide oxidation of pyrite surface (py-S-S) oxidation proceeds in steps that depend on the pH (Rimstidt and Vaughan, 2003). During pyrite oxidation, the disulfide group becomes electropositive because of the removal of electrons to oxidants at cathodic sites (py-S-S^+^ + e^-^). This leads to protonation of the outer S atom with negative dipole ends of water molecules to form py-S-S-OH, which undergoes further removal of electrons and nucleophilic attacks with water to form py-S-SO_3_ (Rimstidt and Vaughan, 2003). At high pH, the terminal S-SO_3_ completely ionizes and the sulfur entity is released as aqueous S_2_O_3_^2-^. At low pH, as is the case in the present study, protons are retained (py-S-SO_3_H), causing electron transfer into the S-S bond and the outer sulfur gaining more positive charge. Further nucleophilic attacks by water release sulfur as aqueous SO_4_^2-^ (Rimstidt and Vaughan, 2003). Although the above abiotic processes do not fully simulate the biological oxidation mechanism, they indicate the possibility that thiosulfate or sulfate can be the first soluble products based on experimental conditions.

From an electrochemical point of view, pyrite oxidation corresponding to a minimum and maximum oxidation state can be described by the following half reactions (Wei and Osseo-Asare, 1997):

(6) $${\mathrm{FeS}}_{2}\to {\mathrm{Fe}}^{2+}+\;2S^{0}+2e^{-}$$

(7) $${\mathrm{FeS}}_{2}+8H_{2}O\to {\mathrm{Fe}}^{3+}+\;2{\mathrm{SO}}_{4}{}^{2-}+16H^{+}+15e^{-}$$

As Fe^2+^ is released into the solution and oxidized to Fe^3+^ (Equations 1 and 2), 14 electron transfers are involved in the oxidation of the sulfur entity by Fe^3+^. The oxidation state of all pyrite oxidation intermediates and products are within the limits of 0 and +6 as defined by Equations 6 and 7.

Electrochemical probes can be effective tools to monitor the pyrite oxidation process. Electrodes with fixed small pyrite grains have been used to investigate the electrochemical behavior of pyrite during its oxidation under biotic and/or abiotic conditions (Mustin et al., 1992, 1993; Toniazzo et al., 1999; Liu et al., 2011b). In addition to basic electrochemical parameters of the process, the increased rate of pyrite oxidation in the presence of bacteria have been clearly demonstrated. Elemental sulfur formation under abiotic conditions was established (Liu et al., 2011a; Nicol et al., 2013). Compared to the electrodes with sulfide grains, the electrodes based on a single crystal provide a low reaction rate and thus a low current. However, its surface is better related to the natural pyrite and the sulfide property is not affected by preparation procedures of the grain electrodes. This system has been used to study electrochemical pyrite oxidation under conditions of changed applied potential (Holmes and Crundwell, 2000). The results showed that the reaction kinetics and electrode mixed potential were correlated. No products accumulated on the pyrite electrode surface except for a small amount of polysulfides. Holmes and Crundwell (2000) concluded that thiosulfate was not the only source of the end sulfur product and the formation of sulfur and sulfate proceeded by independent pathways. Electrochemical study of the oxidation of a crystal pyrite electrode detected sulfate formation, together with S_8_, at the electrode potential at 0.7–0.8 V and pH 2 (Tu et al., 2017b). Liu et al. (2017) studied the oxidation of pyrite electrode by sessile acidophiles. The bacteria had limited impact on pyrite dissolution at and below the redox potential of 650 mV (near the rest potential), but an increasing redox potential (e.g., spontaneously at the uncontrolled potential) resulted in pyrite dissolution. Holmes et al. (1999) and Fowler et al. (1999) used a crystal pyrite electrode to investigate bacterial pyrite oxidation under constant solution conditions maintained at the controlled redox potential. They concluded that bacteria in the biofilm decreased the pH at the electrode surface, followed by a decrease in the mixed potential as an impulse to increase the rate of oxidative dissolution of the pyrite crystal electrode. Electrodes based on the pyrite crystals have also been used to describe pyrite surface colonization by the sulfur-oxidizer *Acidithiobacillus thiooxidans* (González et al., 2012; Lara et al., 2012).

The main advantage of pyrite electrodes is that the current corresponds to electron transfers from the pyrite surface to the oxidant in the solution through the solid-liquid interface. The oxidation state for the identification of the first released sulfur species during pyrite oxidation can be determined by this way. Thus the process parameters and the oxidation mechanism are not affected by other redox reactions in the solution. Soluble sulfur intermediates may be formed by marginal side reactions or secondary reactions in the solution, but without direct relationship to the dominant oxidation mechanism. Chemical determination of intermediates by itself does not demonstrate directly the process mechanism at a level of pyrite surface-solution interface. We have previously applied this approach to characterize the mechanism of arsenopyrite oxidation, based on compact electrodes containing arsenopyrite as a polycrystalline aggregate (Zeman et al., 1995). The oxidation and reduction rates were dependent on the potential of the electrode and were established at the rest potential value when the rate of both the oxidation and reduction were the same. Under these steady-state conditions, the anodic and cathodic currents were compensated directly on the surface of the mineral electrode (Zeman et al., 1995): *i_cat_* = *i_anod_* = *i_0_*, where *i_0_* is the exchange current. The exchange current density *j_0_* obtained from *i_0_ (j_0_ = i_0_/A, A* is the electrode area) is directly proportional to the rate of pyrite oxidation because of Faraday’s law, which shows a relationship between the amount of released iron and the exchange current:

(8) $$N=Q/(nF)=i_{0}t/(nF)$$

where *N* is the molar amount of released iron at the exchanged current *i_0_, Q* is the charge passed across the mineral-solution interface, *t* is the time, *n* is the number of exchanged electrons (that are consumed by the oxidant for oxidation of one molecule of pyrite), and *F* is the Faraday’s constant. At the steady-state, *i_0_* cannot be directly measured but only evaluated from the polarization curves. When the system is electrically shifted from the steady-state by polarization with outside applied potential, net cathodic or anodic current is obtained according to the direction of polarization.

We have used a compact pyrite electrode based on a pyrite polycrystalline aggregate to monitor bacterial pyrite oxidation. The previous preliminary results (Mandl et al., 1999) indicated sulfate formation upon pyrite biooxidation, based on a limited chemical and electrochemical approach. The kinetic data of ferric iron reduction by pyrite were also used to support the main results but this was deemed a poorly reproducible and unreliable approach. However, those results prompted us to undertake a more detailed electrochemical investigation, supplemented by metabolic studies, to clarify the mechanism of pyrite oxidation. In addition to standard electrochemical parameters, which were used to characterize the process, determination of the number of exchange electrons during spontaneous pyrite oxidation led to another approach to study the process mechanism. The purpose of the present study was to examine sulfate formation as the dominant first aqueous sulfur species of bacterial pyrite oxidation under suitable redox conditions. Thiosulfate, or other sulfur intermediates included in the thiosulfate mechanism, may be formed but as a result of side reactions in pyrite oxidation or secondary reactions in the solution.

## Materials and Methods

### Pyrite Electrode

**FIGURE 1:**
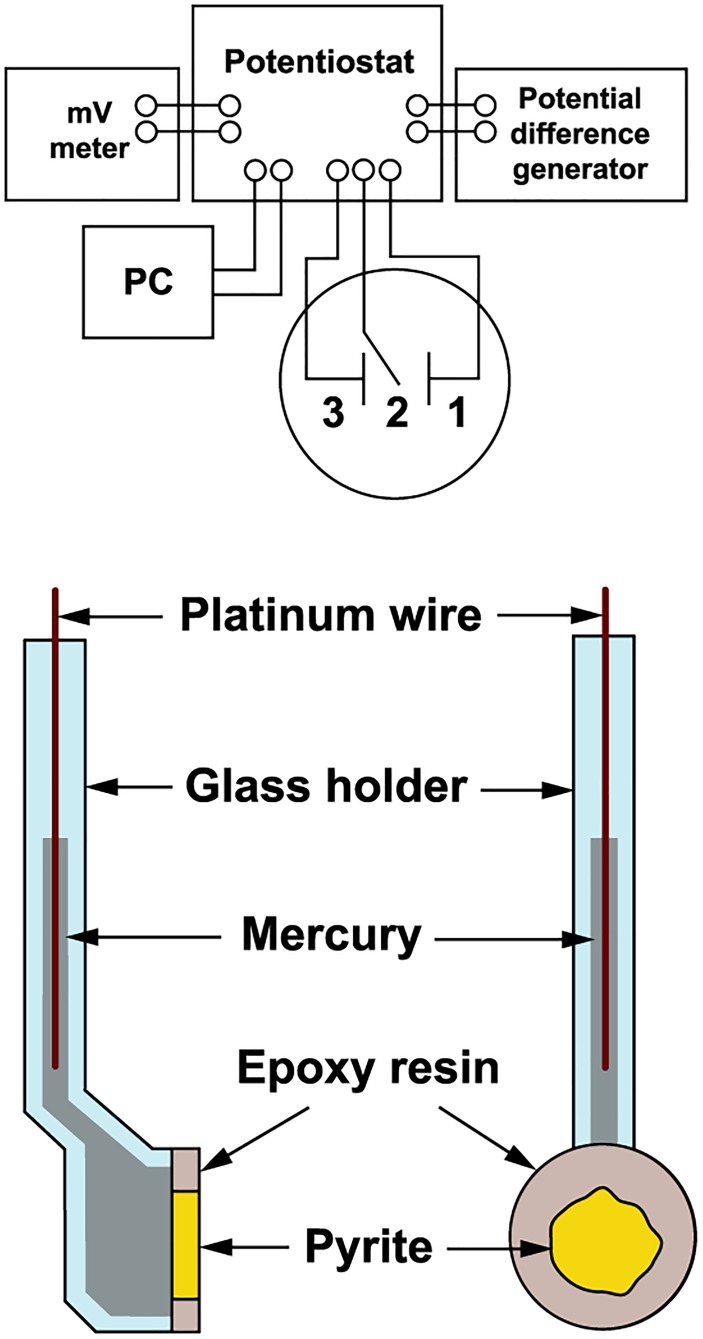
Schematic diagram of the electrochemical arrangement. The pyrite electrode was a working electrode (1), the saturated calomel electrode a reference electrode (2), and the platinum net electrode a counter electrode (3).

The pyrite electrode was used as previously described for arsenopyrite oxidation (Zeman et al., 1995). The scheme of the electrode and measurement arrangement is shown in Figure 1. Polycrystalline pyrite aggregate (from the Zlaté Hory copper-lead-zinc sulfide ore deposits, Czechia) of approximately 1 cm in diameter was sealed in epoxy resin and cut to disks 2.5 cm in total diameter and 2 mm thick. Disks were polished on both sides and mounted with a silicon sealant to special glass holders. The electrical connection between the inner surface of the electrode and the polarization device was created by liquid mercury and platinum wire. The outer surface of pyrite was in direct contact with oxidative solution (in the presence or absence of bacteria). The pyrite surface during the oxidation process was checked by scanning electron microscopy (CamScam, Cambridge Instruments Co., London, United Kingdom).

#### Electrochemical Arrangement

The pyrite electrode was immersed in a cell (50 mL) containing an oxidant (bacteria or pH 1.8 acidified water as a control). Pyrite oxidation without permanent electrode polarization was investigated over a period of 100 days using exchange current density as a direct measure of the reaction rate. The exchange current was represented by the current passing across the mineral-solution interface due to the redox reaction alone. The redox potential (*Eh*) was measured with a combined InLab Redox electrode (Mettler-Toledo AG, Greifensee, Switzerland) using a pH-mV meter PHM 93 (Radiometer, Copenhagen). The rest potential of the pyrite electrode (*Es*) was measured against a saturated calomel electrode using the pH-mV meter. The pyrite electrode was used as a working electrode, the saturated calomel electrode as a reference electrode, and the platinum net electrode as a counter electrode (Figure 1). Polarization of the working electrode was carried out using the potentiostatic unit of a polarographic analyzer (PA3, Laboratory Devices, Prague). The polarization curve used to determine the exchange current was obtained by briefly polarizing the pyrite electrode within 1 min of each observation. The linear potential sweep rate was 1 mV/s in both cathodic and anodic directions from the rest potential of working electrode, i.e., to -100 and 100 mV overpotentials, respectively.

#### Exchange Current Density and the Number of Exchanged Electrons

To obtain the value of the exchange current density, the Butler-Volmer equation (Equation 9) was fitted to the experimental polarization curves *j* = f(*E*) (after the current was converted to current density):

(9) $$j=j_{0}\{e^{\left[(E-Es)/b_{a}\right]}-e^{\left[-(E-Es)/b_{c}\right]}\}$$

where *j* is the net current density depending on the applied potential *E, j_0_* is the exchange current density, *b_a_* and *b_c_* are the Tafel’s constants for anodic and cathodic reactions, respectively, *Es* is the rest potential of the pyrite electrode at steady-state. Only polarization curves were measured directly. *j_0_* (*i_0_*/A at steady-state, when *E* = *Es*) and the other constants in Equation 9 were obtained numerically from the Equation (9) using the least-squares, non-linear curve fitting. Classical Tafel analysis of polarization curves requires rather high polarization potentials (e.g., Li et al., 2016). In the case of the oxidation mechanism study, the course of the oxidation process could be different from that of the real (unpolarized) state, which might change the reaction mechanism.

The number of exchanged electrons was determined using Equation 10, based on Equation 8:

(10) $$i_{0}=nFdN/dt$$

The rate of pyrite oxidation was expressed as *v* = *dN*/*dt*, based on its proportionality to the rate of formation of aqueous iron that was released from the pyrite electrode during its oxidation. The rate was determined from the experimental time dependence of dissolved iron concentration. A parabola equation was fitted to that dependence *N* = f(*t*) to be differentiated in order to obtain *dN*/*dt*. The values of *i_0_* and *v* in corresponding times provided a linear dependence (Equation 10) with a slope of *nF*, from which the *n* value was obtained.

Standard deviations (SD) of the electrochemical parameters were obtained from triplicates. The second and third samplings were arranged after 30 min from the electrode polarization, when the pyrite electrode was electrochemically fully restored to its original electrochemical state. SD are indicated with vertical bars in the figures. To detect the *n* variability obtained from individual electrodes, the samples of three pyrite electrodes were processed. The total charge *Q* passed across the pyrite-solution interface was used for this case. Equation 11 gives its value:

(11) $$Q=\int_{t_{0}}^{t}i_{0}dt$$

where (*t* – *t_0_*) is the length of investigated period. *Q* was determined using integration of the experimental dependence of *i_0_* on *t* for 100 days and *n* was calculated based on Equation 8 to obtain the *n* value for each electrode:

(12) n=Q/(NF)

where *N* is the end molar amount of released iron corresponding to oxidized pyrite moles during the investigated period of 100 days.

#### Bacteria and Oxidative Solutions

*Acidithiobacillus ferrooxidans* (CCM 4253) was used in this study. The strain has 100% identity with the type strain ATCC 23270^T^ by 16S rRNA gene sequencing (Pokorna et al., 2007). The whole genome sequencing data are available at the GenBank (GCA_003233765.1). Culture conditions for growth on ferrous iron have been previously described (Bouchal et al., 2006). Standard batch cultures were based on mineral salts medium 9K. The culture was harvested by centrifugation (6500 *g* for 30 min), washed and resuspended in water acidified with sulfuric acid to pH 1.8. The final cell number in the suspension of the oxidative solution was 10^9^/mL. The abiotic oxidative solution contained water acidified to pH 1.8.

### Oxidation of the Pyrite Concentrate

Pyrite concentrate (41% Fe, 45% S) with a mean particle diameter 40 μm was used as described previously (Mandl and Vyskovsky, 1994). Pyrite concentrate (2 g) in mineral salts medium without iron (100 mL) at pH 1.8 was inoculated (10% v/v) with a culture grown on Fe^2+^. The culture was incubated in 500-mL shake flasks on a rotary shaker at 28°C. To study cellular ATP in the attached cells, the culture was grown in 1 L mineral salts medium without iron, supplemented with 20 g pyrite concentrate, in a vessel with agitation at 200 rpm and aeration with filter-sterilized air 0.5 L/min at 28°C. Abiotic process of tetrathionate degradation (suspension of 100 mL containing 1 mM tetrathionate, 2 g pyrite concentrate and 1 mM ferric iron) was assessed on a rotary shaker as described for the culture conditions.

#### Analytical Procedures

Fe^2+^ was determined colorimetrically with *o*-phenanthroline (Tamura et al., 1974), Fe^3+^ spectrophotometrically at 300 nm (Mandl and Novakova, 1993), and total iron by ICP spectrometry (iCAP 6500, Thermo Fisher Scientific, Waltham, MA, United States). The number of bacteria was determined using a Cyrus chamber and an optical microscope BX50 (Olympus, Tokyo). Tetrathionate was determined by mass spectrometry (6224 TOF LC/MS, Agilent Technologies, Santa Clara, CA, United States). Cellular ATP content of planktonic and attached cells was determined as previously described using a bioluminescence assay (Pakostova et al., 2013a). Sufficient amount of attached cells for ATP determination were obtained using the detachment protocol developed previously for cells growing with elemental sulfur (Pakostova et al., 2013b). Differences in the ATP contents were tested by the *t*-test.

#### RNA Isolation and Real-Time PCR

The cultivation conditions were similar to those of the 1-L culture vessel except for a 10-L culture vessel volume and agitation at 400 rpm. For reverse-transcription quantitative PCR (RT-qPCR), biomass was harvested by filtration (Whatman filter paper) to remove pyrite followed by centrifugation (14,000 *g* for 10 min at 4°C) at the beginning (time zero) and each third day of bacterial growth. The planktonic cell pellets were washed with mineral salts 9K medium and frozen at -70°C. Total RNA was isolated from three biological replicates of 200 mL using the TRI Reagent (Sigma-Aldrich) and DNA was removed using DNase I (Thermo Scientific) according to the manufacturer’s guidelines. The expression of the selected genes (the tetrathionate hydrolase *tetH*, the subunit d*oxDA* of the thiosulfate-quinone oxidoreductase complex, and the subunit *hdrA* of the heterodisulfide oxidoreductase complex) involved in energy metabolism of reduced inorganic sulfur compounds was determined by RT-qPCR as previously described (Kucera et al., 2013) using gene-specific oligonucleotides, the sequences of which were shown earlier (Kucera et al., 2016). The relative expression level (*R*) of each gene was normalized using the housekeeping gene (*alaS*) to facilitate evaluation of gene expression in relation to the internal standard by the ΔΔCT method (Pfaffl, 2001; Nieto et al., 2009). The normalized values at different time points during the growth periods were compared with those obtained at time zero (when the inoculum was added). A twofold deviation in the expression ratio was regarded as an indicator of significant differential gene expression and each gene expression value was evaluated by the *t*-test, with the significance threshold set at *P* < 0.05.

## Results

### The Oxidation of Pyrite Electrode

**FIGURE 2:**
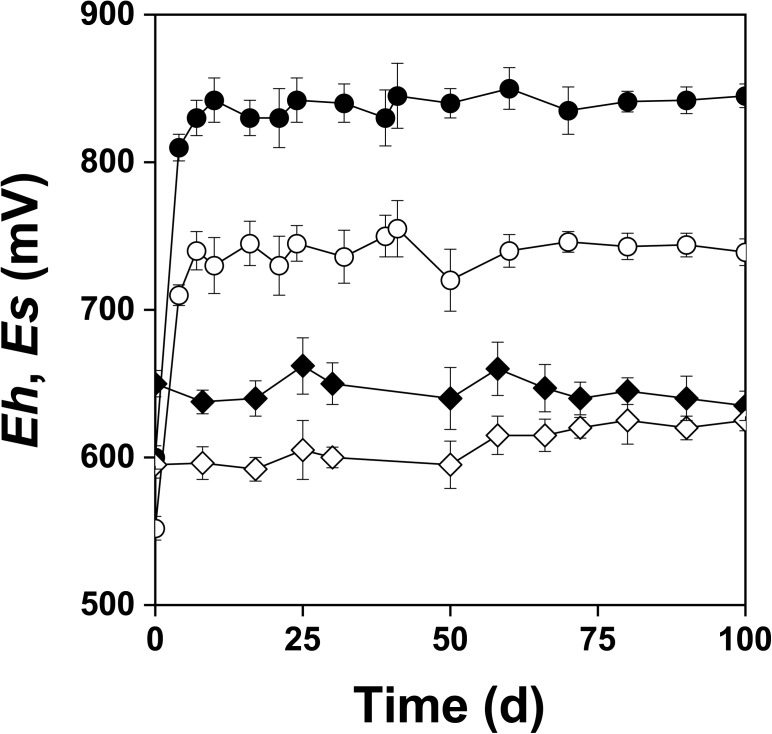
Changes in redox (*Eh*) and rest (*Es*) potentials (mV vs. SHE) during oxidation of the pyrite electrode in the presence (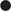, *Eh*; 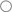, *Es*) and absence (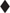, *Eh*; 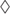, *Es*) of bacteria. The abiotic control solution was water acidified with sulfuric acid to pH 1.8.

**FIGURE 3:**
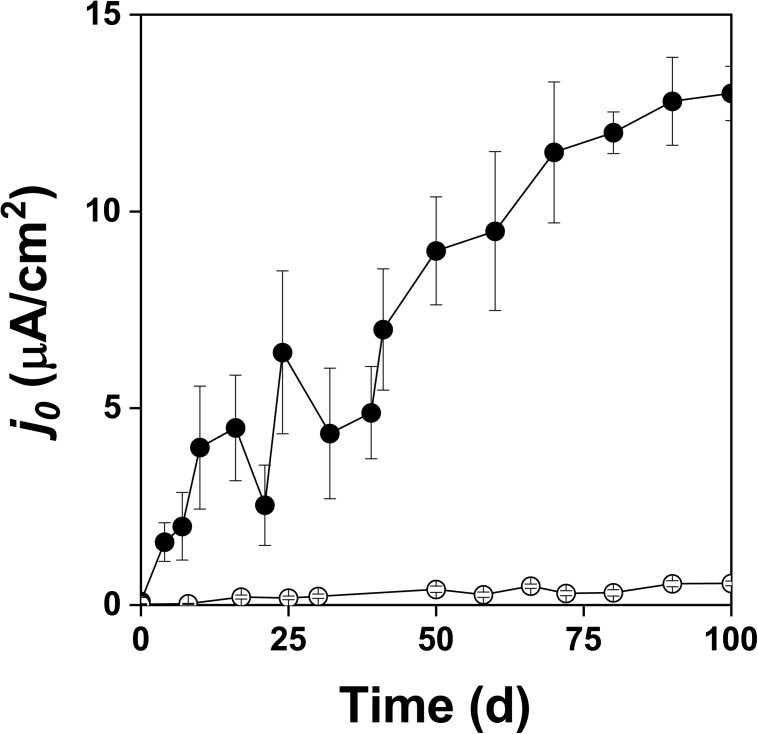
Exchange current density (*j_0_*) during oxidation of the pyrite electrode in the presence (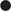) and absence (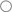) of bacteria.

**FIGURE 4:**
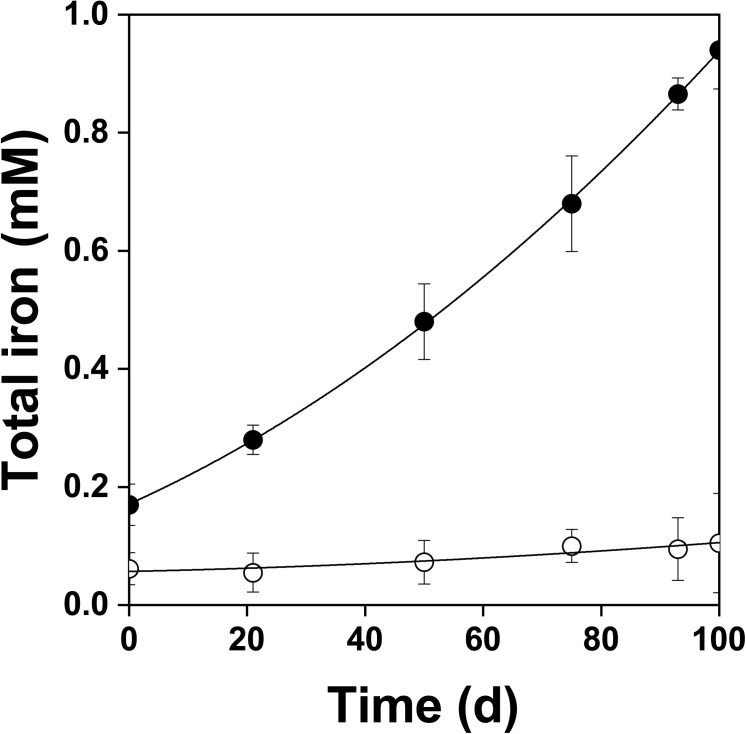
Changes in total dissolved iron concentration during oxidation of the pyrite electrode in the presence (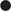) and absence (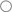) of bacteria. In contrast to the abiotic control, dissolved iron was in the ferric form in the presence of bacteria. The difference between the concentrations of total iron and Fe^3+^ was insignificant, *P* > 0.05.

**FIGURE 5:**
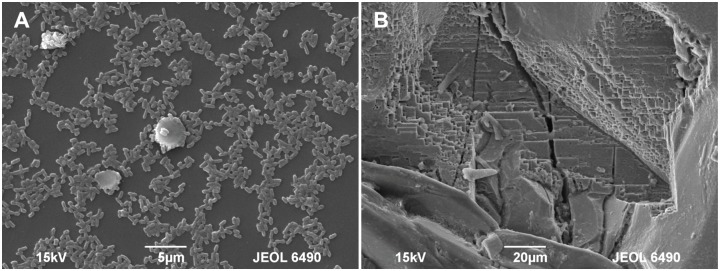
SEM micrographs of the pyrite electrode surface. **(A)** Attached bacteria after 10 days of contact; **(B)** Pronounced corrosion of the pyrite surface after 60 days of contact.

Figure 2 shows the changes of *Eh* and *Es* over time during the oxidation of the pyrite electrode. The difference between the *Eh* and *Es* represents a driving force for pyrite oxidation and its value for bacterial oxidation was clearly higher compared to the abiotic control. Exchanged current density (*j_0_*) increased with time (Figure 3) and was directly related to the 100 days time course of bacterial pyrite oxidation and the dissolution of iron (Figure 4). Due to the high bacterial activity, dissolved iron was in the ferric form (the amount of iron precipitates was negligible) and the difference between the concentrations of total iron and Fe^3+^ was insignificant (*P* > 0.05). Figure 3 shows that the *j_0_* value was almost negligible in the absence of bacteria, which is in agreement with the low amount of iron released abiotically from pyrite during 100 days (Figure 4). SEM micrographs showed attached bacteria on pyrite surface (Figure 5A). The number of cells per unit area changed with time and the specific site of observation of pyrite surface. Figure 5A represents an area of high cell density. Corroded sites of the pyrite surface were more common during later phases of the time course when the concentration of ferric iron had increased. Sites of intensive corrosion of the pyrite surface without bacterial presence were also evident (Figure 5B), indicating the etching of pyrite surface by ferric iron, the dominant corrosive agent in this process.

#### The Number of Exchanged Electrons

**FIGURE 6:**
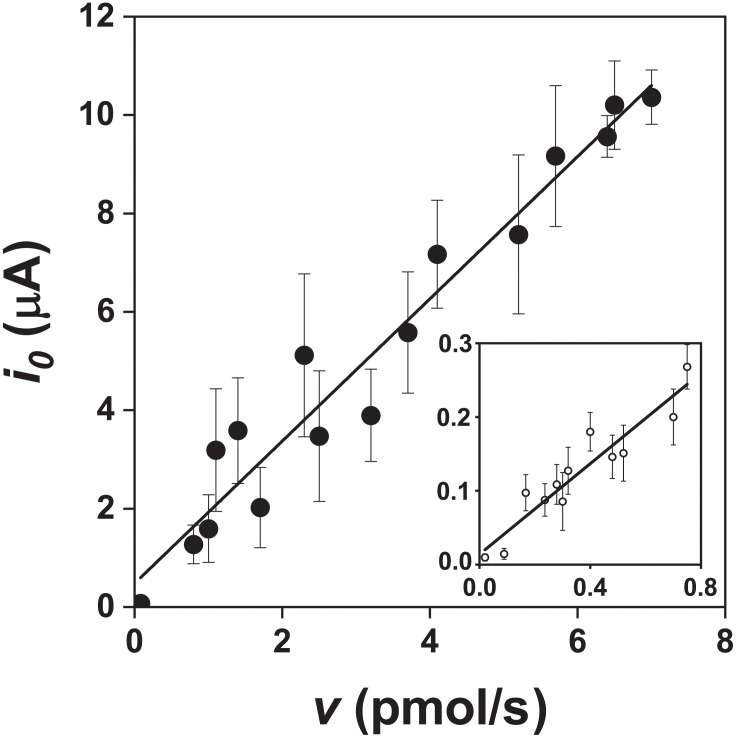
Relationship between the exchange current (*i_0_*) and pyrite oxidation rate (*v*, expressed as the rate of iron released) during oxidation of the pyrite electrode in the presence (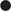) and absence (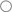) of bacteria (abiotic control solution was water at pH 1.8). The *v* values were obtained from the data in Figure 4 after fitting a parabola equation to the experimental data of the iron concentration time course and its differentiation (based on Equation 10). Ninety-nine confidence intervals for the numbers of exchanged electrons per one molecule of pyrite were 14.5 ± 2.7 and 3.1 ± 1.1 in the presence and absence of bacteria, respectively.

The slopes calculated from the data in Figure 6 were 1.40 ± 0.26 × 10^6^ C/mol in the presence of bacteria and 0.30 ± 0.11 × 10^6^ C/mol in the abiotic control (99% confidence intervals). The corresponding numbers of exchanged electrons (*n*) for the oxidation of the pyrite electrode were calculated from the data in Figure 6 and Equation 10. The *n* values per one molecule of pyrite were 14.5 ± 2.7 in the presence of bacteria and 3.1 ± 1.1 in the abiotic control (99% confidence intervals). As the difference between 14.5 and 14 (the maximum *n* value for the process at the pyrite-solution interface) was insignificant (*P* > 0.05), the *n* value indicates dominant sulfate formation according to Equation 1. The *n* value for thiosulfate formation is 6 per one molecule of pyrite, which is significantly different from *n* = 14.5 (*P* < 0.01). The abiotic control (*n* = 3.1) included elemental sulfur formation (*n* = 2), a dominant but slow oxidation process in the absence of bacteria.

The confidence interval estimates calculated from the *n* values from three individual electrodes reflected slightly higher experimental errors than those obtained from the data in Figure 6 because of another arrangement and evaluation. Based on Equations 11 and 12, the following *n* values were obtained for the biotic process: 13.8, 14.2, and 15.0. The 99% confidence interval was 14.3 ± 3.5. The *n* values from the control electrodes in the absence of bacteria were 2.2, 2.3 and 2.6. The 99% confidence interval was 2.4 ± 1.2. These results are in agreement with the above interpretation of the numbers of exchanged electrons during oxidation of pyrite electrode in the presence or absence of bacteria.

#### Oxidation of the Pyrite Concentrate

##### The pyrite culture and cellular ATP content

**FIGURE 7:**
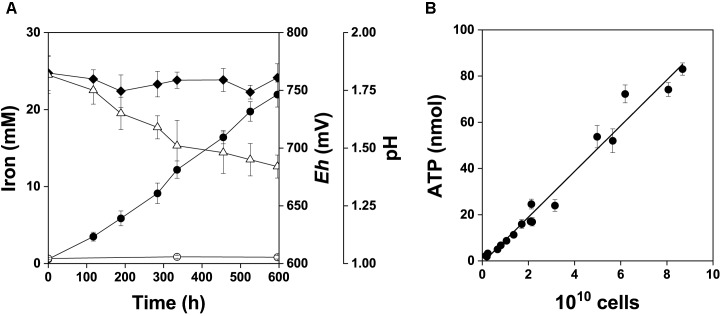
Bacterial oxidation of pyrite concentrate, 2% w/v pulp density. The data are shown for the active growth phase. A short lag phase and the beginning of the stationary phase are excluded from this graph and were not used in data analysis. **(A)** Fe^2+^ (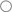), Fe^3+^ (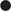), redox potential (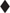, *Eh*, mV vs. SHE) and pH (Δ). **(B)** Relationship between the total amount of ATP and total cell number in the pyrite culture, *r* = 0.988.

*A. ferrooxidans* was grown in mineral salts medium amended with 2% w/v pyrite concentrate in order to supplement the pyrite electrode data with culture-based data. Changes in pH, *Eh*, and dissolved iron species in the pyrite culture over the 25 days time course are shown in Figure 7A. The relationship between the total amount of ATP and total cell numbers in the pyrite culture is shown in Figure 7B. The pyrite culture was in the maximum growth and iron oxidation phase. These culture conditions kept iron dissolved from pyrite in the predominant Fe^3+^ form and redox potential continually high in parallel with decreasing pH. The mean cellular ATP content was determined for planktonic cells sampled from active growth phase. Based on the slope in Figure 7B, this value was 0.99 ± 0.12 amol ATP per cell (99% confidence interval). The difference between this ATP content and 1.16 amol ATP of cells oxidizing Fe^2+^ (Pakostova et al., 2013a) was insignificant (*P* > 0.05), indicating that planktonic cells in the pyrite culture used Fe^2+^ as the source of energy, in keeping with the concentration of Fe^3+^ increasing with time. The corresponding values for cells growing with elemental sulfur, thiosulfate (Pakostova et al., 2013b) and tetrathionate (unpublished data) are 0.33, 0.63, and 0.5 amol ATP per planktonic cell, respectively. The differences between 0.99 amol ATP and the above range of values for the oxidation of sulfur substrates were significant (*P* < 0.01). Thus Fe^2+^ was predominantly oxidized by planktonic cells during pyrite oxidation without a dominant contribution of direct bacterial oxidation of sulfur compounds. Five samples of planktonic and attached cells were used to test the cellular ATP content during the active growth phase with maximum oxidation activity. The results for planktonic and attached cells were 1.10 ± 0.19 and 1.14 ± 0.27 amol ATP per cell (99% confidence intervals), respectively, and this was an insignificant difference (*P* > 0.05).

###### Abiotic degradation of tetrathionate and speciation of sulfur compounds

The chemical stability of tetrathionate was tested under our experimental conditions of pyrite concentrate oxidation. During 25 days of contact in pH 1.8 acidified water containing 2% pyrite and 1 mM Fe^3+^, 29% of the initial 1 mM tetrathionate was degraded abiotically. This result was very similar to that reported by Schippers et al. (1996).

**FIGURE 8:**
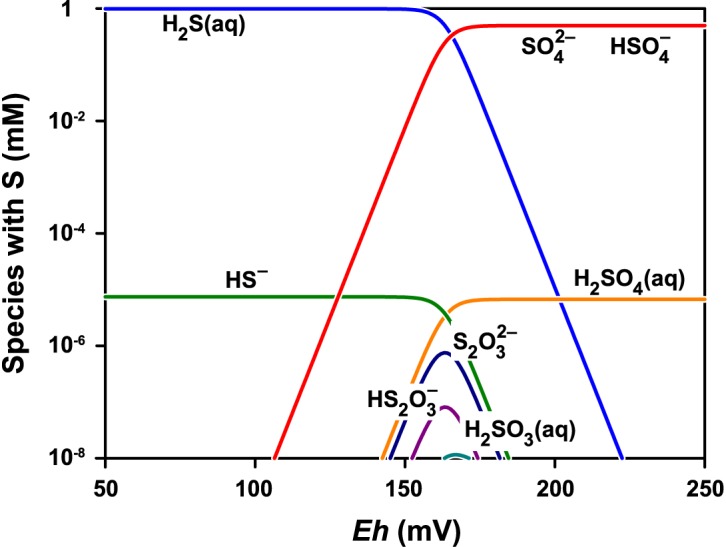
Speciation of sulfur at pH 1.8 and total concentration of sulfur species normalized to 1 mM. The speciation model contained 24 species including thiosulfate S_2_O_3_^2-^, pentathionate S_5_O_6_^2-^, dithionite S_2_O_4_^2-^, sulfite SO_3_^2-^, and dithionate S_2_O_6_^2-^. Only the species with concentration greater than 10^-11^ M are shown. The GWB software was used to obtain the model for the actual conditions (Bethke and Yeakel, 2018).

Figure 8 shows the stability diagram of sulfur species as a function of *Eh* at pH 1.8. The main sulfur species are SO_4_^2-^ and HSO_4_^-^ under the experimental conditions. The concentrations of the other sulfur species with lower oxidation states are more than a million times lower and are limited to a relatively narrow range of low redox potentials.

##### Relative gene expression

**FIGURE 9:**
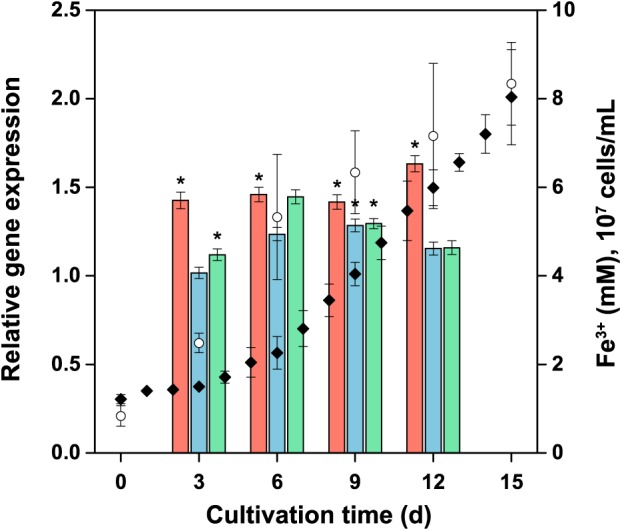
Expression of selected genes involved in energy metabolism of reduced inorganic sulfur compounds during pyrite concentrate oxidation by *A. ferrooxidans* planktonic cells. The culture was generated using ferrous iron-grown cells as the inoculum, with an initial number of 10^7^ cells per mL. Columns: *tetH* 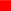; *doxDA* 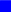; and *hdrA* 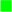 represent the average relative expression normalized to the endogenous control (*alaS*) for each culture period (ΔCTs). Symbols: Fe^3+^ (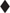); cell number (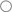). Error bars indicate the standard deviation (*n* = 3); ^∗^, significant difference (*P* < 0.05).

The relative expression of the tetrathionate hydrolase (*tetH*), the subunit *doxDA* of the thiosulfate-quinone oxidoreductase complex, and the subunit *hdrA* of the heterodisulfide oxidoreductase complex was monitored at transcript levels in the planktonic biomass during the bacterial oxidation of pyrite (Figure 9). No significant change (*R* < 2 and *P* > 0.05) in the expression was observed for these genes. Thus, the expression of these sulfur metabolism genes in *A. ferrooxidans* planktonic cells appeared to be at a low basal level in the pyrite culture.

## Discussion

There is a general consensus that pyrite oxidation takes place by an indirect mechanism, which can be described by Equations 1 and 2. The total overall pyrite oxidation, which includes both the chemical and bacterial reactions given by the above two equations, can be described with Equation 13:

(13) $$4{\mathrm{FeS}}_{2}+15O_{2}+2H_{2}O\to 4{\mathrm{Fe}}^{3+}+8{\mathrm{SO}}_{4}{}^{2-}+4H^{+}$$

At the low pH, which was used under pyrite oxidation conditions, ferric and sulfate ions are in the solution although subsequent reactions (e.g., Fe^3+^ hydrolysis and formation of complexes) may continue with time. The indirect process is based on non-contact or contact bacterial leaching, illustrating the activity of planktonic or attached bacteria that oxidize predominantly Fe^2+^ in the solution or at the pyrite surface to regenerate Fe^3+^ in the solution (Vera et al., 2013). Thiosulfate has generally been recognized as the first soluble sulfur intermediate. Pyrite oxidation may take place under various conditions that can modify the oxidation mechanism, resulting in changes in the formation of intermediate sulfur compounds. The key factor to influence the reaction course is the combination of the pH and redox conditions. The effect of redox conditions, associated with ferric iron regeneration by *A. ferrooxidans*, on the oxidation state of pyrite oxidation products was demonstrated in this study.

The results of the present study indicated that sulfate is the first aqueous sulfur species formed in the bacterial oxidation of the pyrite electrode under suitable redox conditions, because about 14 electrons per pyrite molecule were exchanged in the oxidation. Although the *n* value in the presence of bacteria is related to sulfate formation as the dominant process, the formation of intermediates such as elemental sulfur or thiosulfate from minor side reactions is conceivable although electrochemically not detected within the experimental error. Because of the 99% confidence interval of the *n* value (14.5 ± 2.7), minor formation of elemental sulfur or thiosulfate could decrease the *n* value below 14, but only by a small fraction within the confidence interval width. Thus, the effect of the formation of elemental sulfur or thiosulfate was negligible or marginal and without a serious impact on the final evaluation, because there was a highly significant difference between 14.5 and 2 (elemental sulfur formation) or 6 (thiosulfate formation). In addition to the dominant sulfate formation, such minor formation affecting slightly and insignificantly the *n* value would only result from side reactions, proceeding in parallel with sulfate formation. Substantial formation of sulfur intermediates with a lower oxidation state should transiently, but significantly, decrease the *n* value below 14 until completion of sulfate formation. This was not apparent from the estimated *n* values. In fact, the stability diagram confirmed that the possibility of the presence of other sulfur species in addition to sulfate is extremely low.

Pyrite oxidation by *Leptospirillum ferrooxidans* results in slightly higher formation of partially oxidized sulfur species that tend to persist unlike in the case of pyrite oxidation by *A. ferrooxidans* (Schippers et al., 1996; Schippers and Sand, 1999). These aqueous sulfur oxyanions as well as elemental sulfur are the result of side reactions but they accumulate if the sulfur-oxidizers are absent. They have been detected under experimental conditions that were clearly different from those of the present study. Even with dominant sulfate, a low level of only partially oxidized aqueous sulfur compounds may suppress the redox potential of the solution phase, which may further result in only a partial oxidation of pyritic sulfur. By comparison to *L. ferrooxidans*, the activity of *A. ferrooxidans* produces more acid because of sulfate formation. Active *A. ferrooxidans* helps maintain a suitable redox potential range for the complete oxidation of pyrite, because it also oxidizes elemental sulfur and partially oxidized oxyanions that may marginally form in side or secondary reactions. In contrast, *L. ferrooxidans* must be accompanied with sulfur-oxidizing bacteria to reach a similar redox potential range. It is another example to illustrate that no universal mechanism exists under all ambient redox and acidity conditions.

Our electrochemical observations are in agreement with sulfate formation during the chemical oxidation of pyrite at low pH (McKibben and Barnes, 1986). In contrast to studies demonstrating thiosulfate as the first intermediate, the formation of sulfate as the first aqueous sulfur species has been derived from theoretical consideration. The key factor of sulfate formation is based on the effect of acidic conditions due to H^+^ participation in the pyrite surface oxidation, while thiosulfate is released into the solution at higher pH (Rimstidt and Vaughan, 2003). Our results also emphasize key importance of the redox conditions on the oxidation state of the products. The high redox potential kept by bacterial activity resulted in sulfate formation as the first dominant, aqueous sulfur species. The isotopic composition of sulfate confirmed the stoichiometry of the thiosulfate mechanism (Balci et al., 2007). This was based on intermediate sulfite oxidation by oxygen, although there are more reactions consuming oxygen in the thiosulfate mechanism (polythionate oxidation, and especially respiration with sulfur intermediates in the case of active sulfur-oxidizing bacteria). A major difference compared to our conditions is the pH range. Balci et al. (2007) used the pH range from about 3.0 to 2.2, which represents a one order magnitude lower H^+^ concentration as compared to our conditions. A relevant comparison at more depth is not feasible because of the lack of details on redox conditions in experiments reported by Balci et al. (2007).

The redox end rest potentials directly influence the product oxidation state. Our experimental conditions included high redox potential due to the high ratio of the predominant redox couple Fe^3+^/Fe^2+^ as the result of sustained bacterial activity. Liu et al. (2017) noted the importance of the difference between the redox and rest potentials for pyrite dissolution to soluble iron and sulfate. Our data showed how the different redox conditions established with the *Eh* and *Es* values at the same initial pH resulted in sulfate formation in the presence of bacteria or elemental sulfur formation in the abiotic control without bacteria. The rates were also very different. Elemental sulfur formation has also been often observed in various phases of pyrite oxidation (Liu et al., 2011a; Nicol et al., 2013).

In general, electron transfers involving more than two e^-^ at each step are not probable. The oxidation of pyritic sulfur to sulfate therefore involves multiple steps (Wei and Osseo-Asare, 1997), possible up to seven to account for transfer of the 14 e^-^ from the disulfide entity. Under a large difference between the *Eh* and *Es*, which is the driving force of the oxidation process maintained by bacterial iron-oxidizing activity, successive electron transfers take place in the pyrite-solution interface. Sulfate as the final oxidation product is released into the solution. The same principle should be applicable to thiosulfate formed in pyrite oxidation, although six e^-^ transfers per pyrite molecule may include fewer steps.

We focused attention to the phase of maximum cell growth and oxidation activity. Negative changes in bacterial activities during pyrite oxidation, such as cell inactivation, inhibition or substrate limitation, may impact the intensity of iron oxidation and thus Fe^3+^ formation, thereby decreasing the *Eh* and changing the redox conditions and the oxidation mechanism.

Schippers et al. (1996) reported that both the biotic and abiotic course of reactions involved the thiosulfate mechanism. One of the key steps in the thiosulfate mechanism is tetrathionate hydrolysis. Slow abiotic decomposition of tetrathionate was detected under pyrite catalysis to form elemental sulfur and sulfate as well as traces of polythionates. About 25% of the tetrathionate was degraded abiotically in 19 days in the presence of pyrite (Schippers et al., 1996). Under our experimental conditions, about 29% of tetrathionate was degraded during 25 days. The very slow rate of abiotic degradation of tetrathionate cannot serve as the only route to form sulfate during bacterial pyrite oxidation. In addition to the iron-oxidizing activity, the sulfur-oxidizing activity of *A. ferrooxidans* is also necessary to oxidize tetrathionate and other sulfur intermediates to sulfate unless the pathway is mediated by the preponderance ferric iron and the dominant iron-oxidizing activity of bacteria. Complete pyrite oxidation (Equation 13) in the presence of bacteria has been reported, involving concurrent Fe^3+^ and SO_4_^2-^ formation in accordance with the reaction stoichiometry (e.g., Janiczek et al., 1998).

In addition to electrochemical data, we could not confirm sulfur-oxidizing activity of *A. ferrooxidans* in the pyrite culture. No discernible difference was observed in the cellular ATP content between planktonic and attached bacteria. The cellular ATP content is specific for the substrate and corresponded to Fe^2+^ oxidation in this case. The oxidation of elemental sulfur, thiosulfate, or tetrathionate could be excluded by virtue of the specific cellular ATP content. The cellular ATP data do not exclude a marginal utilization of sulfur substrates that may appear as a result of side reactions. Both the ATP data and electrochemical results indicated that iron oxidation during the active pyrite oxidation phase was a dominant process in both planktonic and attached cells.

Due to importance of tetrathionate in the thiosulfate mechanism and its availability for planktonic cells, gene expression of the tetrathionate hydrolase was investigated. Thiosulfate and elemental sulfur, the other typical sulfur substrates that may appear during tetrathionate hydrolysis, should be accessible for the corresponding enzymes in the planktonic cells in contrast to sulfur compounds formed on the pyrite surface. Expression of the selected genes representing several sulfur metabolism pathways was monitored as potential markers of the thiosulfate mechanism in planktonic cells under defined conditions. Vera et al. (2009) showed elevated expression of sulfur metabolism genes in sessile *A. ferrooxidans* cells and an increased transcript level of ferrous iron metabolism genes in planktonic cells after 4 days of pyrite oxidation. The endpoint study observed global transcriptomics and proteomics response at a very early process phase, whereas we focused on transcript levels of three selected genes throughout the process. We demonstrated no significant upregulation of selected sulfur metabolism genes in planktonic cells in the pyrite culture over the entire time course. This finding is also in agreement with the short-term (4 days) study reported by Vera et al. (2009), who indicated that pyrite oxidation releases soluble Fe^2+^, which is oxidized by planktonic cells. Insoluble sulfur compounds are also formed on pyrite surface and this fraction is oxidized by sessile cells. It is conceivable that this scenario occurs rather at the beginning of the process, when sessile bacteria start the oxidation on pyrite surface using the exopolymer layer containing complexed Fe^3+^ (Gehrke et al., 1998; Rohwerder et al., 2003). The activity of attached cells to release the initial amount of iron into the solution can be related to the decreasing pH on the pyrite surface (Fowler et al., 1999; Holmes et al., 1999). Sulfur compounds from this initial response may be precursors to several reactions typical in the thiosulfate mechanism. In contrast to thiosulfate, which is oxidized by Fe^3+^ under catalysis of the pyrite surface (Schippers et al., 1996), tetrathionate formed from thiosulfate is a relatively abundant and stable compound at low pH. Its biochemical hydrolysis in the solution may be an important marker of the thiosulfate mechanism, which also includes the oxidation of elemental sulfur and thiosulfate. Thus, the overexpression of the corresponding genes should be expected in a predominantly planktonic *A. ferrooxidans* culture growing with pyrite. However, such results were not observed, although the low basal-level expression may indicate that the planktonic cells were capable of rapid metabolic switch (i.e., adaptation to different substrates) upon changes in experimental condition.

Once iron is released from pyrite into the solution and bacteria regenerate Fe^3+^, the *Eh* increases and pyrite is oxidized according to Equation 1 at low pH especially. Distinct corrosion of the pyrite electrode surface at the sites without the occurrence of bacteria confirmed that Fe^3+^ may be the main oxidant in agreement with Equation 1. During the active oxidation phase, the main role of both planktonic and sessile bacteria is the oxidation of Fe^2+^ at low pH to maintain favorable redox potential conditions, which results in sulfate formation as the first aqueous sulfur species.

## Conclusion

The mechanism of pyrite oxidation by ferric iron to sulfate includes formation of various sulfur intermediates based on experimental conditions. In contrast to the previously reported thiosulfate mechanism, this study demonstrated sulfate as the first aqueous sulfur species formed in the bacterial pyrite oxidation under suitable redox conditions. Pyrite oxidation by *A. ferrooxidans* at pH 1.8 was investigated using a polycrystalline aggregate pyrite electrode. The number of exchanged electrons per pyrite molecule was determined to be close to 14, consistent with dominant sulfate formation. The formation of aqueous sulfur intermediates, if any, is attributed to side reactions associated with pyrite oxidation and secondary reactions in the solution. The impact on the determined number of exchanged electrons by intermediates and products from side reactions could not be discerned within experimental error. Sulfate as the first aqueous sulfur species was formed only under iron-oxidizing activity of *A. ferrooxidans*, re-oxidizing Fe^2+^ to Fe^3+^ and thus sustaining high redox potential. Elemental sulfur formation was detected under the control conditions without bacteria. Iron-oxidizing activity as a dominant process in both planktonic and attached cells oxidizing pyrite concentrate was deduced from the cellular ATP contents, which depends on the specific substrate oxidation. The mechanism based on predominantly ferric-iron mediated pyrite oxidation was in agreement with the absence of significant upregulation of three essential sulfur metabolism genes in planktonic cells. The lack of specific upregulation of the *tetH* gene indicated no or negligible bacterial activity on tetrathionate, the key soluble intermediate of the thiosulfate mechanism. Based on all the results, only marginal amount of intermediate sulfur compounds may be formed as side reactions during active growth and pyrite oxidation phases. The potential presence of tetrathionate, elemental sulfur or thiosulfate in the pyrite culture did not evoke gene upregulation under the described oxidation conditions. Their unchanged basal levels were deemed sufficient for the cell to process marginal amounts of these sulfur compounds. Thiosulfate and polythionates may be more dominant intermediates under different redox and pH conditions in keeping with previous studies of the thiosulfate mechanism. The combination of the degree of acidity and redox conditions represents a key factor that influences the reaction course and the mechanism of bacterial pyrite oxidation.

## Author Contributions

SB performed the electrochemical part of results. MM conceived and designed the project, participated in the result evaluation, and wrote the manuscript. JZ constructed the electrochemical tools and participated in evaluation and text revision of this section. JK performed the gene expression experiments and participated in the manuscript preparation. EP performed determination of cellular ATP contents. OJ performed MS analyses of abiotic tetrathionate degradation. OT provided edits and contributed to data discussion.

## Conflict of Interest Statement

The authors declare that the research was conducted in the absence of any commercial or financial relationships that could be construed as a potential conflict of interest.

## References

[B1] Balci N. Shanks W. C. Mayer B. Mandernack K. W. (2007). Oxygen and sulfur isotope systematics of sulfate produced by bacterial and abiotic oxidation of pyrite. *Geochim. Cosmochim. Acta* 71 3796–3811. 10.1016/j.gca.2007.04.017

[B2] Banerjee I. Burrell B. Reed C. West A. C. Banta S. (2017). Metals and minerals as a biotechnology feedstock: engineering biomining microbiology for bioenergy applications. *Curr. Opin. Biotechnol.* 45 144–155. 10.1016/j.copbio.2017.03.009 28371651

[B3] Bartakova I. Kummerova M. Mandl M. Pospisil M. (2001). Phytotoxicity of iron in relation to its solubility conditions and the effect of ionic strength. *Plant Soil* 235 45–51. 10.1023/A:1011854031273

[B4] Bethke C. M. Yeakel S. (2018). *The Geochemist’s Workbench^®^, Release 11. GWB Essentials Guide*. Champaign, IL: Aqueous Solutions LLC.

[B5] Bigham J. M. Nordstrom D. K. (2000). Iron and aluminum hydroxysulfates from acid sulfate waters. *Rev. Mineral. Geochem.* 40 351–403. 10.2138/rmg.2000.40.7

[B6] Blowes D. W. Ptacek C. J. Jambor J. L. Weisener C. G. Paktunc D. Gould W. D. (2014). “The geochemistry of acid mine drainage,” in *Treatise on Geochemistry*, 2nd Edn Vol. 11 eds Holland H. D. Turekian K. K. (Amsterdam: Elsevier), 131–190. 10.1016/B978-0-08-095975-7.00905-0

[B7] Bouchal P. Zdráhal Z. Helánová S. Janiczek O. Hallberg K. B. Mandl M. (2006). Proteomic and bioinformatic analysis of iron- and sulfur-oxidizing *Acidithiobacillus ferrooxidans* using immobilized pH gradients and mass spectrometry. *Proteomics* 6 4278–4285. 10.1002/pmic.200500719 16807941

[B8] Descostes M. Vitorge P. Beaucaire C. (2004). Pyrite dissolution in acidic media. *Geochim. Cosmochim. Acta* 68 4559–4569. 10.1016/j.gca.2004.04.012

[B9] Dold B. (2014). Evolution of acid mine drainage formation in sulphidic mine tailings. *Minerals* 4 621–641. 10.3390/min4030621 20711792

[B10] Fowler T. A. Holmes P. R. Crundwell F. K. (1999). Mechanism of pyrite dissolution in the presence of *Thiobacillus ferrooxidans*. *Appl. Environ. Microbiol.* 65 2987–2993. 10388693 10.1128/aem.65.7.2987-2993.1999 PMC91446

[B11] Gehrke T. Telegdi J. Thierry D. Sand W. (1998). Importance of extracellular polymeric substances from *Thiobacillus ferrooxidans* for bioleaching. *Appl. Environ. Microbiol.* 64 2743–2747. 9647862 10.1128/aem.64.7.2743-2747.1998 PMC106458

[B12] González D. M. Lara R. H. Alvarado K. N. Valdez-Pérez D. Navarro-Contreras H. R. Cruz R. (2012). Evolution of biofilms during the colonization process of pyrite by *Acidithiobacillus thiooxidans*. *Appl. Microbiol. Biotechnol.* 93 763–775. 10.1007/s00253-011-3465-2 21773763

[B13] Hedrich S. Guézennec A. G. Charron M. Schippers A. Joulian C. (2016). Quantitative monitoring of microbial species during bioleaching of a copper concentrate. *Front. Microbiol.* 7:2044. 10.3389/fmicb.2016.02044 28066365 PMC5167697

[B14] Hipsey M. R. Salmon S. U. Mosley L. M. (2014). A three-dimensional hydro-geochemical model to assess lake acidification risk. *Environ. Model. Softw.* 61 433–457. 10.1016/j.envsoft.2014.02.007

[B15] Holmes P. R. Crundwell F. K. (2000). The kinetics of the oxidation of pyrite by ferric ions and dissolved oxygen: an electrochemical study. *Geochim. Cosmochim. Acta* 64 263–274. 10.1016/S0016-7037(99)00296-3

[B16] Holmes P. R. Fowler T. A. Crundwell F. K. (1999). The mechanism of bacterial action in the leaching of pyrite by *Thiobacillus ferrooxidans*. *J. Electrochem. Soc.* 146 2906–2912. 10.1149/1.1392027

[B17] Janiczek O. Mandl M. Ceskova P. (1998). Metabolic activity of *Thiobacillus ferrooxidans* on reduced sulfur compounds detected by capillary isotachophoresis. *J. Biotechnol.* 61 225–229. 10.1016/S0168-1656(98)00043-1

[B18] Johnson D. B. (2012). Geomicrobiology of extremely acidic subsurface environments. *FEMS Microbiol. Ecol.* 81 2–12. 10.1111/j.1574-6941.2011.01293.x 22224750

[B19] Kaksonen A. H. Morris C. Rea S. Li J. Wylie J. Usher K. M. (2014). Biohydrometallurgical iron oxidation and precipitation: part I—effect of pH on process performance. *Hydrometallurgy* 147–148, 255–263. 10.1016/j.hydromet.2014.04.016

[B20] Kucera J. Bouchal P. Lochman J. Potesil D. Janiczek O. Zdrahal Z. (2013). Ferrous iron oxidation by sulfur-oxidizing *Acidithiobacillus ferrooxidans* and analysis of the process at the levels of transcription and protein synthesis. *Antonie Van Leeuwenhoek* 103 905–919. 10.1007/s10482-012-9872-2 23291738

[B21] Kucera J. Pakostova E. Lochman J. Janiczek O. Mandl M. (2016). Are there multiple mechanisms of anaerobic sulfur oxidation with ferric iron in *Acidithiobacillus ferrooxidans*? *Res. Microbiol.* 167 357–366. 10.1016/j.resmic.2016.02.004 26924114

[B22] Lara R. H. García-Meza J. V. Cruz R. Valdez-Pérez D. González I. (2012). Influence of the sulfur species reactivity on biofilm conformation during pyrite colonization by *Acidithiobacillus thiooxidans*. *Appl. Microbiol. Biotechnol.* 95 799–809. 10.1007/s00253-011-3715-3 22113561

[B23] Li L. Polanco C. Ghahreman A. (2016). Fe(III)/Fe(II) reduction-oxidation mechanism and kinetics studies on pyrite surfaces. *J. Electroanal. Chem.* 774 66–75. 10.1016/j.jelechem.2016.04.035

[B24] Liu C. Jia Y. Sun H. Tan Q. Niu X. Leng X. (2017). Limited role of sessile acidophiles in pyrite oxidation below redox potential of 650 mV. *Sci. Rep.* 7:5032. 10.1038/s41598-017-04420-2 28694428 PMC5504038

[B25] Liu Y. Dang Z. Lu G. Wu P. Feng C. Yi X. (2011a). Utilization of electrochemical impedance spectroscopy for monitoring pyrite oxidation in the presence and absence of *Acidithiobacillus ferrooxidans*. *Miner. Eng.* 24 833–838. 10.1016/j.mineng.2011.03.002

[B26] Liu Y. Dang Z. Wu P. X. Lu J. Shu X. Zheng L. (2011b). Influence of ferric iron on the electrochemical behavior of pyrite. *Ionics* 17 169–176. 10.1007/s11581-010-0492-4

[B27] Luther G. W. (1987). Pyrite oxidation and reduction: molecular-orbital theory considerations. *Geochim. Cosmochim. Acta* 51 3193–3199. 10.1016/0016-7037(87)90127-X

[B28] Mandl M. Novakova O. (1993). An ultraviolet spectrophotometric method for the determination of oxidation of iron sulfide minerals by bacteria. *Biotechnol. Tech.* 7 573–574. 10.1007/BF00156331

[B29] Mandl M. Vyskovsky M. (1994). Kinetics of arsenic(III) oxidation by iron(III) catalysed by pyrite in the presence of *Thiobacillus ferrooxidans*. *Biotechnol. Lett.* 16 1199–1204. 10.1007/BF01020851

[B30] Mandl M. Zeman J. Bartakova I. Vesela H. (1999). “Pyrite biooxidation: Electrochemical and kinetic data,” in *Biohydrometallurgy and the Environment Toward the Mining of the 21st Century, Part A*, eds Amils R. Ballester A. (Elsevier: Amsterdam), 423–429.

[B31] McKibben M. A. Barnes H. L. (1986). Oxidation of pyrite in low temperature acidic solutions: rate laws and surface textures. *Geochim. Cosmochim. Acta* 50 1509–1520. 10.1016/0016-7037(86)90325-X

[B32] Moses C. O. Nordstrom D. K. Herman J. S. Mills A. L. (1987). Aqueous pyrite oxidation by dissolved oxygen and by ferric iron. *Geochim. Cosmochim. Acta* 51 1561–1571. 10.1016/0016-7037(87)90337-1 18939588

[B33] Mustin C. Berthelin J. Marion P. de Donato P. (1992). Corrosion and electrochemical oxidation of a pyrite by *Thiobacillus ferrooxidans*. *Appl. Environ. Microbiol.* 58 1175–1182. 16348688 10.1128/aem.58.4.1175-1182.1992 PMC195571

[B34] Mustin C. de Donato P. Berthelin J. Marion P. (1993). Surface sulfur as promoting agent of pyrite leaching by *Thiobacillus ferrooxidans*. *FEMS Microbiol. Rev.* 11 71–78. 10.1016/0168-6445(93)90026-6

[B35] Nicol M. Miki H. Basson P. (2013). The effects of sulphate ions and temperature on the leaching of pyrite. 2. dissolution rates. *Hydrometallurgy* 133 182–187. 10.1016/j.hydromet.2013.01.009

[B36] Nieto P. A. Covarrubias P. C. Jedlicki E. Holmes D. S. Quatrini R. (2009). Selection and evaluation of reference genes for improved interrogation of microbial transcriptomes: case study with the extremophile *Acidithiobacillus ferrooxidans*. *BMC Mol. Biol.* 10:63. 10.1186/1471-2199-10-63 19555508 PMC2713239

[B37] Nuñez H. Moya-Beltrán A. Covarrubias P. C. Issotta F. Cárdenas J. P. González M. (2017). Molecular systematics of the genus *Acidithiobacillus*: insights into the phylogenetic structure and diversification of the taxon. *Front. Microbiol.* 8:30 10.3389/fmicb.2017.00030 PMC5243848 28154559

[B38] Pakostova E. Mandl M. Omesova Pokorna B. O. Diviskova E. Lojek A. (2013a). Cellular ATP changes in *Acidithiobacillus ferrooxidans* cultures oxidizing ferrous iron and elemental sulfur. *Geomicrobiol. J.* 30 1–7. 10.1080/01490451.2011.636790

[B39] Pakostova E. Mandl M. Tuovinen O. H. (2013b). Cellular ATP and biomass of attached and planktonic sulfur-oxidizing *Acidithiobacillus ferrooxidans*. *Process. Biochem.* 48 1785–1788. 10.1016/j.procbio.2013.07.026

[B40] Pfaffl M. W. (2001). A new mathematical model for relative quantification in real-time RT-PCR. *Nucleic Acids Res.* 29:e45 10.1093/nar/29.9.e45 PMC55695 11328886

[B41] Pokorna B. Mandl M. Borilova S. Ceskova P. Markova R. Janiczek O. (2007). Kinetic constant variability in bacterial oxidation of elemental sulfur. *Appl. Environ. Microbiol.* 73 3752–3754. 10.1128/AEM.02549-06 17449698 PMC1932669

[B42] Quatrini R. Johnson B. D. (2018). Microbiomes in extremely acidic environments: functionalities and interactions that allow survival and growth of prokaryotes at low pH. *Curr. Opin. Microbiol.* 43 139–147. 10.1016/j.mib.2018.01.011 29414445

[B43] Rimstidt J. D. Vaughan D. J. (2003). Pyrite oxidation: a state-of-the-art assessment of the reaction mechanism. *Geochim. Cosmochim. Acta* 67 873–880. 10.1016/S0016-7037(02)01165-1

[B44] Rohwerder T. Gehrke T. Kinzler K. Sand W. (2003). Bioleaching review part A: progress in bioleaching: fundamentals and mechanisms of bacterial metal sulfide oxidation. *Appl. Microbiol. Biotechnol.* 63 239–248. 10.1007/s00253-003-1448-7 14566432

[B45] Schippers A. Hedrich S. Vasters J. Drobe M. Sand W. Willscher S. (2014). “Biomining: metal recovery from ores with microorganisms,” in *Geobiotechnology I. Advances in Biochemical Engineering/Biotechnology* Vol. 141 eds Schippers A. Glombitza F. Sand W. (Berlin: Springer), 1–47. 10.1007/10_2013_216 23793914

[B46] Schippers A. Jozsa P. G. Sand W. (1996). Sulfur chemistry in bacterial leaching of pyrite. *Appl. Environ. Microbiol.* 62 3424–3431. 16535406 10.1128/aem.62.9.3424-3431.1996 PMC1388944

[B47] Schippers A. Rohwerder T. Sand W. (1999). Intermediary sulfur compounds in pyrite oxidation: implications for bioleaching and biodepyritization of coal. *Appl. Microbiol. Biotechnol.* 52 104–110. 10.1007/s002530051495

[B48] Schippers A. Sand W. (1999). Bacterial leaching of metal sulfides proceeds by two indirect mechanisms via thiosulfate or via polysulfides and sulfur. *Appl. Environ. Microbiol.* 65 319–321. 9872800 10.1128/aem.65.1.319-321.1999 PMC91023

[B49] Sethurajan M. van Hullebusch E. D. Nancharaiah Y. V. (2018). Biotechnology in the management and resource recovery from metal bearing solid wastes: recent advances. *J. Environ. Manage.* 211 138–153. 10.1016/j.jenvman.2018.01.035 29408062

[B50] Tamura H. Goto K. Yotsuyanagi T. Nagayama M. (1974). Spectrophotometric determination of iron(II) with 1,10-phenanthroline in the presence of large amounts of iron(III). *Talanta* 21 314–318. 10.1016/0039-9140(74)80012-3 18961462

[B51] Toniazzo V. Lazaro I. Bernard B. Mustin C. (1999). Bioleaching of pyrite by *Thiobacillus ferrooxidans*: fixed grains electrode to study superficial oxidized compounds. *C. R. Acad. Sci. Paris. Ser. IIA Earth Planet. Sci.* 328 535–540. 10.1016/S1251-8050(99)80135-9

[B52] Tu Z. Guo C. Zhang T. Lu G. Wan J. Liao C. (2017a). Investigation of intermediate sulfur species during pyrite oxidation in the presence and absence of *Acidithiobacillus ferrooxidans*. *Hydrometallurgy* 167 58–65. 10.1016/j.hydromet.2016.11.001

[B53] Tu Z. Wan J. Guo C. Fan C. Zhang T. Lu G. (2017b). Electrochemical oxidation of pyrite in pH 2 electrolyte. *Electrochim. Acta* 239 25–35. 10.1016/j.electacta.2017.04.049

[B54] Vera M. Rohwerder T. Bellenberg S. Sand W. Denis Y. Bonnefoy V. (2009). Characterization of biofilm formation by the bioleaching acidophilic bacterium *Acidithiobacillus ferrooxidans* by a microarray transcriptome analysis. *Adv. Mater. Res.* 71-73 175–178.

[B55] Vera M. Schippers A. Sand W. (2013). Progress in bioleaching: fundamentals and mechanisms of bacterial metal sulfide oxidation-part A. *Appl. Microbiol. Biotechnol.* 97 7529–7541. 10.1007/s00253-013-4954-2 23720034

[B56] Wei D. Osseo-Asare K. (1997). Semiconductor electrochemistry of particulate pyrite: mechanisms and products of dissolution. *J. Electrochem. Soc.* 144 546–553. 10.1149/1.1837446

[B57] Werner A. Meschke K. Bohlke K. Daus B. Haseneder R. Repke J. U. (2018). Resource recovery from low-grade ore deposits and mining residuals by biohydrometallurgy and membrane technology potentials and case studies. *ChemBioEng. Rev.* 5 6–17. 10.1002/cben.201700019

[B58] Wu X. Wong Z. L. Sten P. Engblom S. Österholm P. Dopson M. (2013). Microbial community potentially responsible for acid and metal release from an Ostrobothnian acid sulfate soil. *FEMS Microbiol. Ecol.* 84 555–563. 10.1111/1574-6941.12084 23369102 PMC3732381

[B59] Zeman J. Mandl M. Mrnuštíková P. (1995). Oxidation of arsenopyrite by *Thiobacillus ferrooxidans* detected by a mineral electrode. *Biotechnol. Tech.* 9 111–116. 10.1007/BF00224408 19229526

[B60] Zheng K. Li H. Wang L. Wen X. Liu Q. (2017). Pyrite oxidation under simulated acid rain weathering conditions. *Environ. Sci. Pollut. Res.* 24 21710–21720. 10.1007/s11356-017-9804-9 28762047

